# Barrier-Strengthening Effects of Cannabidiol on Porcine Peyer’s Patches

**DOI:** 10.3390/ijms26073360

**Published:** 2025-04-03

**Authors:** Elisa Boehm, Linda Droessler, Marie-Luise Vollstaedt, Laura Stein, Salah Amasheh

**Affiliations:** 1 Institute of Veterinary Physiology, School of Veterinary Medicine, Freie Universität Berlin, 14163 Berlin, Germany; elisa.boehm@fu-berlin.de (E.B.); linda.droessler@fu-berlin.de (L.D.); marie-luise.vollstaedt@fu-berlin.de (M.-L.V.); laura.stein@fu-berlin.de (L.S.); 2 Marine Science Station, The University of Jordan, Aqaba Branch, Aqaba 77110, Jordan

**Keywords:** Peyer’s patch, epithelial barrier, cannabidiol, tight junctions, claudins

## Abstract

Cannabidiol (CBD), a major non-psychoactive cannabinoid of the *Cannabis sativa* L. plant, has demonstrated anti-inflammatory effects in various studies. However, the therapeutic use of CBD is still limited. Despite its potential, little is known about the molecular mechanisms of CBD on epithelial integrity, particularly concerning effects in native intestinal tissue. To accomplish this, our study aimed to investigate the effects of CBD ex vivo on the follicle-associated epithelium of Peyer’s Patches (PP) and villus epithelium (VE) from porcine intestine. To measure the epithelial barrier, the Ussing chamber technique was employed, followed by immunoblotting and confocal laser-scanning immunofluorescence microscopy of tight junction proteins and specific receptors. The results revealed that CBD significantly strengthens the epithelial barrier of PP by upregulation of sealing tight junction proteins, including occludin, claudin-1, -3, and -7. Additionally, the study showed the potential of CBD to decrease the expression of Tumor necrosis factor alpha (TNFɑ) receptor 1 (TNFR-1) in PP that plays a key role in chronic inflammatory diseases. The study highlights the potential of CBD in the prevention of inflammatory conditions and underlines the important role of PP as a target for bioactive compounds.

## 1. Introduction

The majority of immune cells are located in the gastrointestinal tract, demonstrating that the gut-associated lymphoid tissue (GALT) plays a pivotal role in the immune defense of the body [1]. The GALT is composed of single isolated lymphoid follicles and aggregated follicles called Peyer’s patches (PP) [2].

PP were first described as “distinct aggregated nodules” by Konrad Peyer in 1677 and are typically located in the lamina propria mucosae and submucosae of the small intestine [3,4]. Like other lymph nodes, they consist of a T and a B zone [5]. In regions where the follicles extend into the mucosa, resembling domes, the typical intestinal structures of villi and crypts are absent [6]. The epithelium in this specific location is named follicle-associated epithelium (FAE) and contains specialized microfold cells (M cells) [7,8,9]. These M cells have the function of presenting antigens to the underlying lymph follicles via transcytosis to induce an immune response or tolerance against the antigens [10,11]. Therefore, the paracellular barrier in this region is tighter, preventing an uncontrolled uptake of antigens [12]. However, both villus epithelium (VE) and PP FAE are considered to be leaky epithelia with transepithelial electrical resistance (TER) values below 1000 Ω cm^2^, as their average TER values are 30.4 ± 3.1 Ω cm^2^ and 56.1 ± 3.7 Ω cm^2^, respectively [12,13,14].

For a healthy and intact gut barrier, it is essential for the immune system to distinguish between potential pathogens and beneficial bacterial flora or common food components [15]. The capability of the immune system to balance between a potentially harmful and helpful immune response is crucial to prevent an overactive immune system that could lead to tissue damage [16]. This complex regulation is disturbed in inflammatory bowel diseases (IBDs) such as Crohn’s disease and ulcerative colitis [17,18]. The inappropriate immune response in those chronic diseases leads to a disruption of the epithelial barrier and increased intestinal permeability, which is considered to be the first pathogenetic mechanism in IBDs [19,20]. Tumor necrosis factor alpha (TNFɑ) and its receptors, TNF receptor 1 (TNFR-1) and TNF receptor 2 (TNFR-2), play a crucial role in the disease progression in IBDs [21,22]. The overproduction of TNFɑ leads to cytotoxicity in healthy cells, resulting in tissue damage and exacerbation of symptoms [23,24].

In a previous study conducted by our working group, we demonstrated the beneficial effects of the non-psychoactive phytocannabinoid cannabidiol (CBD) derived from the *Cannabis sativa* plant on the gut barrier by employing the porcine intestinal IPEC-J2 cell line [25]. CBD had a barrier-strengthening effect via upregulation of sealing tight junction (TJ) proteins, and was also found to reduce the expression of the TNFR-1 receptor [25]. In various in vitro and ex vivo studies, CBD already demonstrated its anti-inflammatory effects: In an in vitro study from Aswad et al., CBD extract was shown to downregulate the secretion of pro-inflammatory cytokines, such as interleukin-6 and TNFɑ, in mouse macrophages, human-derived neutrophils stimulated with lipopolysaccharide (LPS), and human-derived T cells activated with anti-CD3 and anti-CD28. Further in vivo studies demonstrated that CBD could reduce pro-inflammatory cytokine levels in mice treated with LPS by peritoneal and intranasal administration [26]. CBD has also been shown to have positive effects on 2,4,6-trinitro-benzene sulfonic acid colitis in a rat model, with less mucosal edema, bleeding, and inflammatory cells [27]. In a few other studies, CBD has also been demonstrated to reduce severity of experimental colitis in rodents [28,29,30,31]. These studies highlight the potential of CBD as an anti-inflammatory drug. However, there is a lack of data from investigations of CBD effects on the porcine intestine, which is more comparable to the human intestine, as well as on the molecular mechanism, particularly regarding TJ proteins, in more complex models than a single cell line.

This study aimed to analyze the effects of CBD on the porcine intestine, focusing on the FAE of lymphatic–active PP and VE, which is involved in resorption. For the analysis, the Ussing chamber technique was employed to measure the TER and paracellular fluxes. After the measurements, the tissue was used for further molecular analyses. Based on our previous study of the effects of CBD on the IPEC-J2 cell line, we intended to gain a deeper understanding of how CBD modulates gut barrier integrity in a more complex ex vivo model.

## 2. Results

### 2.1. Impact of CBD on Transepithelial Barrier Function of Porcine Peyer’s Patch and Villus Epithelium

**Figure 1:**
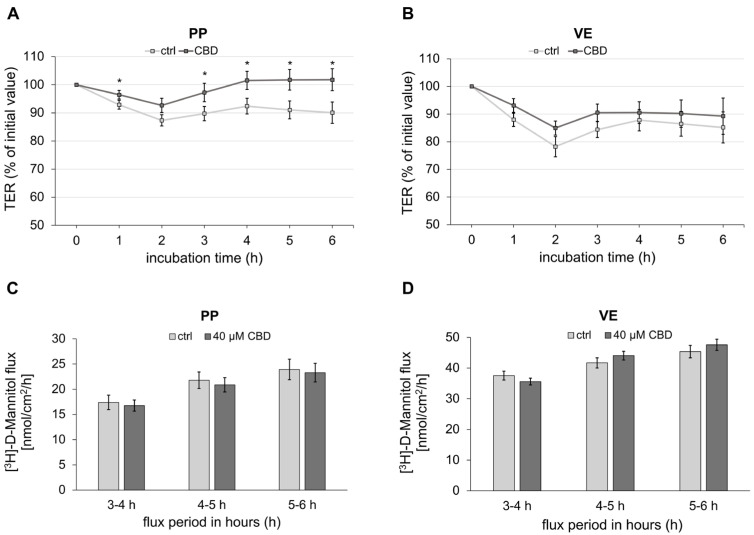
Impact of CBD on transepithelial electrical resistance (TER) and paracellular permeability. (**A**) Change in TER over 6 h of incubation with 40 µM CBD in Peyer’s Patch (PP; Kruskal–Wallis test; * *p* < 0.05; *n* = 25). (**B**) villus epithelium (VE; *n* = 18). (**C**) Paracellular permeability of [^3^H]-D-Mannitol was measured for 3 h after incubation with CBD for 3 h in PP (*n* = 17). (**D**) VE (*n* = 15). Mannitol was added immediately after the first buffer exchange at 2 h, with CBD and ethanol concentrations maintained. Serosal samples were taken every hour starting 1 h after mannitol addition, resulting in three flux periods. Data are presented as percentage of initial resistance ± standard error of the mean (SEM).

To analyze the effects of CBD on the porcine intestine, the transepithelial electrical resistance (TER) was continuously recorded in the Ussing chamber. The incubation of PP with 40 µM of CBD lowered the decay of TER over time compared to the respective controls (1 h: ctrl: 92.90 ± 1.57%, CBD: 96.46 ± 1.53%, *p* < 0.05; 3 h: ctrl: 89.77 ± 2.54%, CBD: 97.24 ± 3.27%, *p* < 0.05; 4 h: ctrl: 92.40 ± 2.78%, CBD: 101.55 ± 3.23%, *p* < 0.05; 5 h: ctrl: 91.06 ± 3.18%, CBD: 101.77 ± 3.67%, *p* < 0.05; 6 h: ctrl: 90.08 ± 3.82%, CBD: 101.78 ± 3.91%, *p* < 0.05; *n* = 25; Figure 1A). In contrast, TER of VE was not significantly affected by incubation with 40 µM of CBD (*n* = 18; Figure 1B).

The flux marker [^3^H]-D-Mannitol was measured from the mucosal to the serosal side to assess the paracellular permeability. Over the entire period of the last 4 h, no significant difference in paracellular permeability could be detected between the control and the incubation with 40 µM CBD in both PP (*n* = 17; Figure 1C) and VE (*n* = 15; Figure 1D).

### 2.2. Immunoblotting

#### 2.2.1. Effects on Tight Junction Proteins

After the incubation experiments, the proteins were extracted to evaluate the effects on the tight junction proteins. The control was set to 100%, and the signal was normalized to the total protein amount.

**Figure 2:**
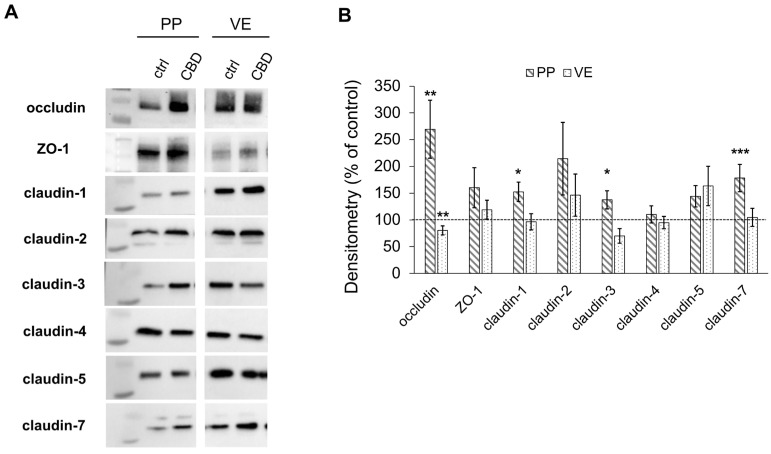
Expression of tight junction proteins after 6 h of incubation with 40 µM of CBD in Peyer’s Patch (PP) and villus epithelium (VE). (**A**) Western blots and (**B**) Densitometry of tight junction proteins (Kruskal–Wallis test, * *p* < 0.05; ** *p* < 0.01; *** *p* < 0.001; *n* = 5–7). The CBD-treated group is compared to controls, which were set to 100% and are shown as the dotted line.

Immunoblotting revealed an increase in the expression of the tight junction protein occludin in PP after incubation with 40 µM of CBD (269.51 ± 54.21%, *p* < 0.01; *n* = 5; Figure 2A,B). In contrast, the expression of occludin decreased in VE when incubated with CBD (80.39 ± 8.39%, *p* < 0.01; *n* = 5; Figure 2A,B). Additionally, the incubation with CBD led to a significantly stronger signal of the sealing TJ proteins claudin 1, 3, and 7 compared to controls in PP (claudin-1: 152.63 ± 18.15%, *p* < 0.05; claudin-3: 137.55 ± 17.05%, *p* < 0.05; claudin-7: 178.25 ± 25.70%, *p* < 0.001; *n* = 7; Figure 2A,B), which was not observed in the VE. The expression of Zonula occludens 1 (ZO-1), claudin-2, -4, and -5 was not significantly affected by incubation with CBD in either PP (*n* = 7) or VE (*n* = 5; Figure 2A,B).

#### 2.2.2. Effects of CBD on the Expression of Cannabinoid and TNF Receptors

**Figure 3:**
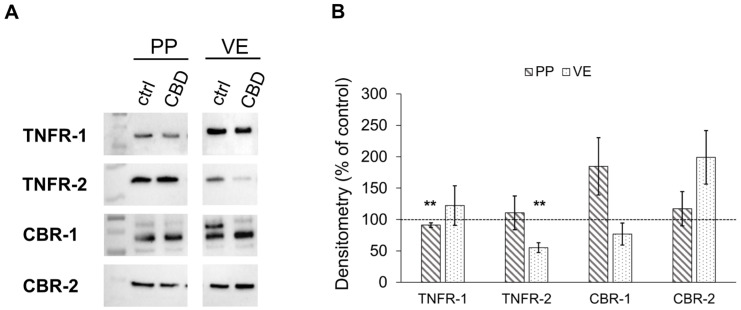
Quantitative analysis of TNF and Cannabinoid receptor expression after 6 h of incubation with 40 µM of CBD in Peyer’s Patch (PP) and villus epithelium (VE). (**A**) Western blots of TNFR-1, TNFR-2, CBR-1, and CBR-2 and (**B**) Densitometry (Kruskal–Wallis test, ** *p* < 0.01; *n* = 4–7). The CBD-treated group is compared to respective controls set to 100%, shown as the dotted line.

The immunoblot analysis of receptor proteins revealed a slight but significant decrease in TNFR-1 receptor expression in PP incubated with 40 µM of CBD compared to the control (91.16 ± 3.75%, *p* < 0.01; *n* = 6; Figure 3A,B). Furthermore, VE showed a decrease in TNFR-2 expression via treatment with CBD (55.23 ± 7.91%, *p* < 0.01; *n* = 5; Figure 3A,B).

On the other hand, no alteration in cannabinoid receptor 1 (CBR-1) and -2 (CBR-2) expression was observed when PP or VE were incubated with 40 µM of CBD compared to controls (*n* = 4–7; Figure 3A,B).

### 2.3. Confocal Laser-Scanning Immunofluorescence Microscopy

**Figure 4:**
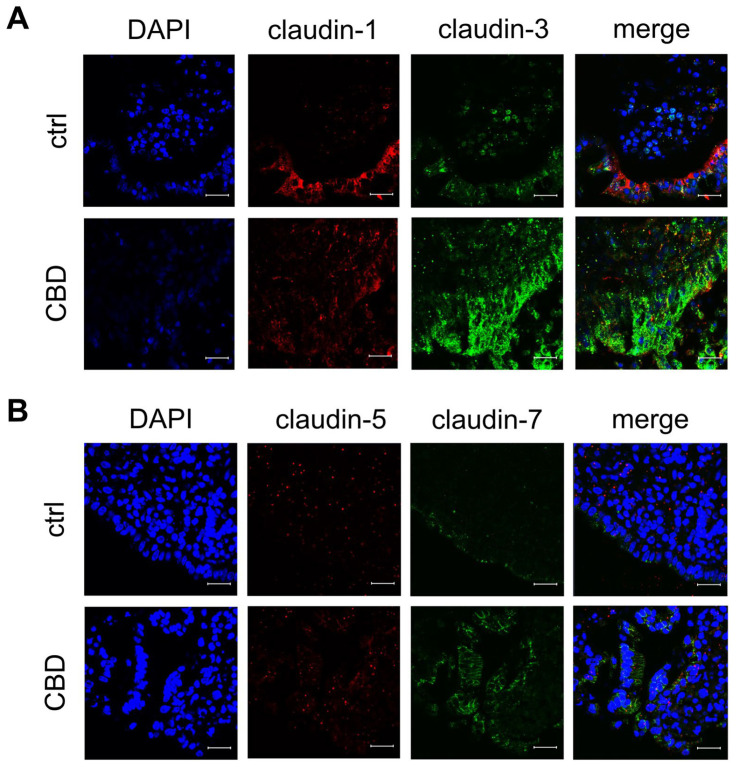
Immunofluorescent staining of follicle-associated epithelium within Peyer’s Patch (PP) after 6 h of incubation with 40 µM of CBD. (**A**) claudin-1 (red) and claudin-3 (green), as well as (**B**) claudin-5 (red) and claudin-7 (green). Nuclei were stained in blue (DAPI; *n* = 7; scale bar: 20 µm; representative images).

**Figure 5:**
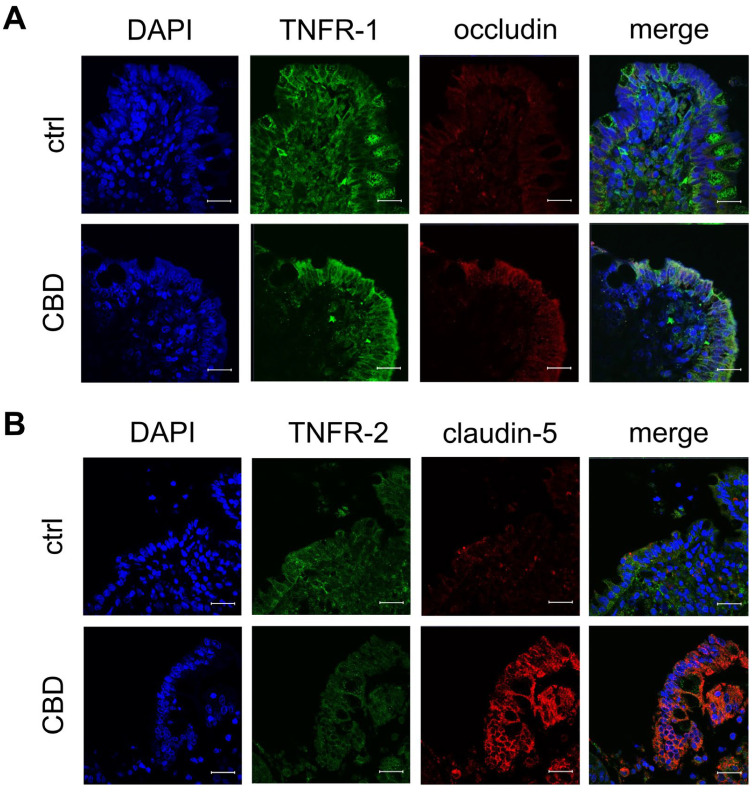
Confocal laser microscopy images of TNF receptors and tight junction proteins in Peyer’s Patch (PP) after incubation with 40 µM of CBD for 6 h. Immunofluorescent staining of (**A**) TNFR-1 (green) and occludin (red), (**B**) TNFR-2 (green) and claudin-5 (red). Nuclei were stained in blue (DAPI; scale bar: 20 μm; *n* = 7; representative images).

To confirm that the TJ proteins and the receptors were not only quantitatively upregulated but also localized in the functionally relevant membrane areas, immunohistochemistry was performed. In accordance with immunoblot signals, PP incubated with 40 µM of CBD showed more intensive junctional signals for the tightening claudins-3 (green; *n* = 7; Figure 4A), -5 (red; *n* = 7; Figure 4B), and -7 (green; *n* = 7; Figure 4B) in the follicle-associated epithelium compared to controls. Furthermore, PP incubated with 40 µM of CBD shows a more intense apical signal for occludin than the control (red; *n* = 7; Figure 5A).

In PP control tissue, the signal for TNFR-1 is more evenly distributed across the whole epithelium, whereas in PP treated with 40 µM of CBD, the signal is more apically located (green; *n* = 7; Figure 5A). In contrast, the distribution of TNFR-2 is not markedly affected by incubation with CBD (green; *n* = 7; Figure 5B).

### 2.4. Vitality Test for Peyer’s Patch

**Figure 6:**
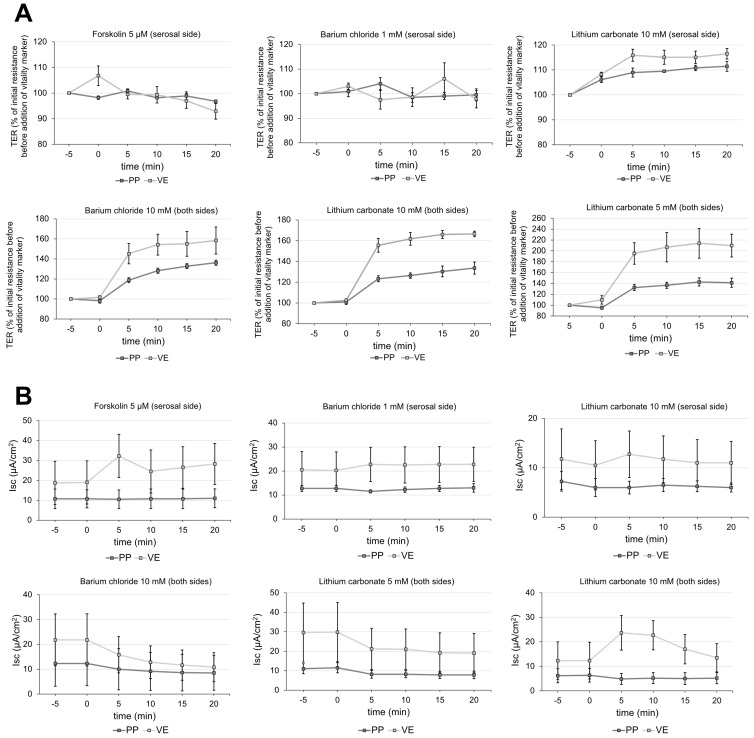
Analysis of different vitality markers for Peyer’s Patch (PP) and villus epithelium (VE). After 3 h [Forskolin 5 µM (serosal side), barium chloride 1 mM (serosal side), and lithium carbonate 10 mM (serosal side)] or after 10 h [barium chloride 10 mM (both sides), and lithium carbonate 5, 10 mM (both sides)] in the Ussing chamber the vitality marker was added, and the (**A**) TER along with the (**B**) short-circuit current (Isc) were recorded for 20 min. The TER value 5 min before addition was set to 100% (*n* = 3–4).

A variety of vitality markers were employed for verification of the vitality of tissue samples. Barium chloride and lithium carbonate added to both sides of the Ussing chamber affected TER of PP and VE (Figure 6A). Since 5 mM of lithium carbonate already showed a marked effect, we decided to use this lower concentration for the experiments (TER before addition: 100%; 20 min after addition: PP: 141.3 ± 8.40%, *p* < 0.01; VE: 209.7 ± 21.02%, *p* < 0.01; *n* = 3; Figure 6A). None of the vitality markers showed significant effects on the short-current circuit (Isc) in PP (Figure 6B).

## 3. Discussion

To date, the only application of CBD (Epidiolex^®^, Jazz Pharmaceuticals, Palo Alto, CA, USA) approved by the Food and Drug Administration (FDA) and the European Medicines Agency (EMA) is for treatment of seizures associated with Lennox–Gastaut syndrome and Dravet syndrome [32,33].

Many studies suggest that CBD can be a promising drug with high potential in multimodal therapies of patients with inflammatory diseases, as it decreases inflammatory cytokines and lowers the activity of immune cells and macrophages [30,34,35]. Despite these results, there is still a lack of information regarding the specific function of CBD on the healthy gut. The effects on porcine intestine, especially on PP, remain mostly unknown. Most studies have been conducted on the Caco-2 cell line or in vivo on rodent models. Therefore, the aim of the study was to shed light on the effects of CBD on a porcine ex vivo model by utilizing intestines from slaughtered pigs, which serves as a valuable alternative to whole animal studies [36,37].

The results show that the TER, representing the barrier function, was prevented from decreasing in PP incubated with CBD compared to the control. This demonstrates the barrier-strengthening effect of CBD, which has recently been described for the first time in our study on the IPEC-J2 cell line [25]. In contrast, CBD did not significantly affect barrier function in VE. Earlier studies have already revealed different barrier properties of PP and VE [12]. Moreover, the FAE that covers PP differs from the VE, as it has a thinner mucus layer leading to a closer contact with the luminal material and is more directly associated with the immune system [38]. This may explain the effects of bioactive compounds such as TNFɑ [39] and caprate [40] on PP. Furthermore, studies from Fujimura et al. suggest that aphthoid ulcers in Crohn’s disease originate from microscopically small disruptions of the FAE due to its closer contact to pathogens [41,42]. Consequently, it can be assumed that barrier-strengthening bioactive substances may also exert their effects within PP.

The prevention of TER decay can be attributed to the upregulation of occludin, claudin-1, claudin-3, and claudin-7 in PP incubated with 40 µM of CBD compared to the control, due to the barrier-strengthening effect of these TJ proteins. Similarly, a study by Li et al. demonstrated an increase in TER by indole-3-propionic acid associated with the upregulation of occludin, claudin-1, and ZO-1 in a Caco-2/HT29 coculture model [43]. In another study involving a bioactive compound, it could relieve dextran sodium sulfate-induced colitis in mice while preventing the negative morphological and functional effects on occludin and ZO-1 [44]. Claudin-1 has also been shown to maintain the epithelial barrier in various studies [45,46]. In diarrhea-predominant irritable bowel syndrome, the barrier-disrupting effect with a decrease in TER can be attributed to a downregulation of claudin-1 [47]. A previous study by Ahmad et al. highlighted the critical role of claudin-3 in promoting colitis and its potential as a target for new therapeutic approaches [48]. Similarly, claudin-7 has been demonstrated to have an important function in maintaining the epithelial barrier, as claudin-7 knock-out mice develop extensive inflammation associated with higher epithelial–mesenchymal transition, which plays a critical role in metastasis and invasion in colorectal cancer [49,50]. Another interesting investigation would have been to compare the protein levels of the 0 h samples with the 6 h samples to compare the effect of CBD over time, but a direct comparison of 6 h controls and 6 h CBD effects might still be considered sufficient as internal controls, given the fact that both tissue samples have been analyzed ex vivo. The functional integration of the TJ proteins occludin and claudin-3, -5, and -7 in the present study was further confirmed through immunohistochemical staining. A clear apical localization within the porcine intestine can be challenging to visualize, but this is a rather common outcome. The immunofluorescence results are only considered a general validation, and are not suitable for quantitative comparison. All these TJ proteins influenced by CBD are highly important for maintaining the intestinal epithelial barrier and therefore represent an essential target for new therapeutic approaches [48].

The low-capacity leak pathway permeability can be assessed by measuring the macromolecular flux of markers across the epithelium, including mannitol, sucrose, inulin, or polymers, such as polyethylene glycols or dextrans, of varying sizes [51]. An impact on the paracellular permeability of [^3^H]-D-Mannitol during incubation with CBD was not detectable in this study. However, it is known that the high-capacity pore pathway and the low-capacity leak pathway, which represents small interruptions in the normally tight TJ contacts, are regulated independently [51,52]. In contrast, in our previous study on IPEC-J2 cells, we observed a decrease in paracellular permeability in cells incubated with CBD after 48 h [25]. The prevention of TER decay compared to the control without effects on paracellular permeability marker flux might indicate an early phase of functional effects in the tissue. Therefore, our current functional data is still in accordance with the previous study in cell culture but limited by experimentally feasible incubation time in the Ussing chamber.

TNFR-1 and -2, the specific receptors for TNFɑ, play a critical role in the progression of IBDs. The TNFR-1 receptor is expressed in most tissues and almost every nucleated cell type, and is regulating inflammation, cell death, and immune response, whereas the TNFR-2 receptor is predominantly found in immune cells and plays a role in cell survival and regeneration [53,54,55]. CBD-incubation led to a slight but significant decrease in the expression of the TNFR-1 compared to the control. These findings are in accordance with previous results of CBD reducing the expression of TNFR-1 [25,56,57]. The downregulation of TNFR-1 without affecting TNFR-2 could be a major advantage in preventive and additive therapies for IBDs. Studies have shown that TNFɑ-induced apoptosis is mediated through the TNFR-1 receptor [22,58,59], while TNFR-2 suppression leads to exacerbation of disease symptoms [60]. However, a significant downregulation of TNFR-2 was observed in VE after incubation with CBD. This is consistent with our findings on IPEC-J2 cells, where the incubation with CBD alone leads to a reduced expression of TNFR-2, while the co-incubation with TNFɑ leads to an increase seen in immunohistochemical staining [25]. This suggests that there are different regulations depending on the basal status of the tissue, such as the apoptotic effect of CBD on tumor cells, which is not observed in healthy cells [61]. Overall, as also shown in previous studies, the behavior of IPEC-J2 cells is more similar to PP than to VE [39,62].

Limitations of the ex vivo Ussing chamber experiments include the interindividual differences between the animals, ranging from different health statuses, diet, and administration of medication to genetic variations in the endocannabinoid system [63,64,65]. In addition, there is the challenge of tissue viability, which comprises identifying a solid vitality marker. Forskolin, which is commonly used as a vitality marker, is expected to increase the transmembrane current (*I*_SC_) as it induces chloride secretion [66]. As this cannot be observed in the PP tissue, we performed Ussing chamber experiments in order to find suitable vitality markers. Barium chloride, as a blocker of potassium channels, led to an increase of the TER [67]. Lithium carbonate was shown to inhibit Na-K ATPase in rat brain synaptosomes [68]. The inhibition of the Na-K ATPase in the intestine could lead to an alteration in the resistance values of the tissue. Furthermore, an inhibition of the Glycogen Synthase Kinase 3 by lithium carbonate could affect the TJ composition and thereby the TER [69]. The effect would be long-term rather than short-term as in our case. Lithium carbonate increased the TER in this study, but as a single parameter of vitality, it is not reliable. This is due to its TER decrease in tissue with very low TER, which is considered non-vital. For this reason, we used the cut-off value from Droessler et al. as a second parameter of vitality [39]. Another possible way lithium carbonate could alter the TER is by affecting the transport of other ions, including sodium and potassium [70]. Thus, the exact mechanism by which lithium carbonate increases the TER needs to be further investigated. Therefore, reliable vitality markers for PP tissue in the Ussing chamber experiments are needed. Nevertheless, the advantages of the ex vivo model are the presence of morphological and physiological determinants, including membrane transporters and nutrient absorption. The Ussing chamber technique enables electrophysiological measurements of tissue samples ex vivo and thus provides deeper insights into membrane properties of the intestinal epithelium [63]. Therefore, ex vivo Ussing chamber experiments can be a helpful model by addressing the current lack of sufficient in vitro PP models to investigate the impact of the mucosal immune system when testing potential new drugs [63,71].

Further investigations might include related phytocannabinoids, other pro-inflammatory agonists, and approaches that more closely match the in vivo properties and allow long-term investigations, such as organoids [65]. To accomplish this, we herewith lay the base for understanding the potentially beneficial intestinal effects of CBD, relevant for epithelial barrier integrity in health and disease.

## 4. Materials and Methods

### 4.1. Tissue Preparation

The tissue was collected from pigs at a slaughterhouse (Lehr- und Versuchsanstalt für Tierzucht und Tierhaltung, Ruhlsdorf, Germany). Samples of ileal and eventually jejunal Peyer’s patch and jejunal villus epithelium were taken from the intestine of slaughtered pigs for the Ussing chamber experiments. For this purpose, the intestine was freed from the serosa and muscularis layer, rinsed with buffer, and immediately placed in ice-cold buffer for transportation.

### 4.2. Buffer

Before transport, the buffer (Table 1) was adjusted to a pH value of approximately 7.4 and gassed with 95% oxygen and 5% carbon dioxide. 

### 4.3. Chemicals

All chemicals were purchased from Carl Roth GmbH, Karlsruhe, Germany, unless otherwise noted. Cannabidiol (CBD; Tocris, Bristol, UK) was dissolved in ethanol to obtain a stock concentration of 40 mM, with a final concentration of 40 µM of CBD in the Ussing chamber. The concentration was selected based on previous studies of different concentrations of CBD in vitro [25]. Ethanol at a final concentration of 0.1% served as control. Lithium carbonate, as a vitality marker, was dissolved in deionized water to obtain a stock concentration of 25 mM.

### 4.4. Ussing Chamber Experiment

The intestine was cut open lengthwise for the Ussing chamber experiments and was mounted into the chamber. After an equilibration period of approximately 45 min, a short-circuit current was applied. The chambers were constantly gassed with 95% oxygen and 5% carbon dioxide at 37 °C. The tissue area measured 0.96 cm^2^. CBD was then added to the mucosal side at 40 µM. The final ethanol concentration was standardized at 0.1%, representing the control. To analyze the effects of CBD under normal conditions, the transepithelial electrical resistance R_t_ [Ω cm^2^] was measured during an incubation time of 6 h. To eliminate interindividual differences in the TER between the animals, the initial values were set to 100% for better comparability. TER values were then compared to the selfsame tissue. A buffer exchange was performed after 2 h incubation time to maintain tissue viability. Immediately after the experiment, tissue samples were cut in half. One half was cryopreserved in liquid nitrogen for protein extraction, and the other half was fixed in 4% paraformaldehyde for 4 h and stored in 25 mM of glycine in PBS with Ca^2+^ and Mg^2+^ containing 0.1% NaN_3_ for immunofluorescence staining until further processing.

### 4.5. Paracellular Flux Measurement

To assess the paracellular flux, 2 μCi [^3^H]-D-Mannitol (PerkinElmer, Waltham, MA, USA) was added to the mucosal compartment. The Ussing chamber experiment was performed as described above. Mannitol was added immediately after the buffer exchange at 2 h. The concentrations of CBD and ethanol were maintained. Samples were collected from the mucosal side at the beginning and at the end of the experiment to calculate the specific activity of the flux marker, using the following Equation (1):

(1) $$\mathrm{specific}\;\mathrm{activity}\;[\mathrm{nmol}]=\frac{mean({counts}_{donor\;side})}{{concentration}_{donor\;side}\times {volume}_{donor\;side}}$$

Samples from the serosal side were then taken every hour, starting one hour after the addition of mannitol, resulting in three flux periods. The volume that was taken for the samples was refilled with fresh buffer containing the respective concentrations of CBD and ethanol. Aquasafe 300 plus liquid scintillation cocktail (Zinsser Analytic, Frankfurt, Germany) was added to all samples and then analyzed by a TriCarb 4910 TR liquid scintillation counter (PerkinElmer, Waltham, MA, USA). With the following equation, the paracellular flux of mannitol was calculated (2):

(2) $$J\;[\mathrm{nmol}\;\times \;\mathrm{cm}{-}^{2}\;\times \;h{-}^{1}]=\frac{{counts}_{t}\times \frac{V_{chamber}}{V_{sample}}-{counts}_{t-1}\times \frac{V_{chamber}-V_{dilution}}{V_{sample}}}{specificactivity\times area\times time}$$

### 4.6. Protein Extraction and Immunoblots

The extraction of the proteins was conducted using RIPA buffer containing 25 mM HEPES pH 7.6, 25 mM NaF, 2 mM EDTA, 1% sodium dodecyl sulfate (10%), H_2_O, and enzymatic protease inhibitors (Complete EDTA-free, Boehringer, Mannheim, Germany), focusing on selfsame tissue samples which have been analyzed ex vivo. The tissue underwent repeated crushing with 2 steel balls, followed by centrifugation to isolate the proteins in the supernatant. Protein quantification was performed by using the Bio-Rad DC Protein Assay (Bio-Rad Laboratories GmbH, Munich, Germany). Immunoblots were carried out as previously described by Boehm et al. (2023) [25].

#### 4.6.1. Tight Junction Proteins

Membranes were incubated with specific antibodies (all from Thermo Fisher Scientific if not declared otherwise, at concentrations according to the manufacturers recommendations) against claudin-1 (cat. #37-4900), claudin-2 (cat. #32-5600), claudin-3 (cat. #34-1700), claudin-4 (cat. #32-9400), claudin-5 (cat. #35-2500), claudin-7 (Abcam, cat. #AB95221), occludin (cat. #33-1500), and ZO-1 (cat. #33-9100).

#### 4.6.2. Cannabinoid and TNF Receptors

To assess the influence of CBD on cannabinoid and TNF receptors, the antibodies (from antibodies-online unless otherwise stated) against CBR-1 (cat. #ABIN3183698), CBR-2 (cat. #ABIN680168), TNFR-1 (Abcam, cat. #AB19139), and TNFR-2 (cat. #ABIN2789622) were used.

### 4.7. Immunohistochemistry

For dehydration, the tissue underwent a treatment with increasing alcohol concentrations (3 × 70% ethanol each for 1 h, 70% overnight, 3 × 80% ethanol each for 1 h, 80% overnight, 90% ethanol for 1 h, 2 × 96% ethanol each for 1 h, 2 × 100% ethanol each for 30 min, Ethanol-Xylol for 40 min) and xylene (2 × 30 min). Tissue samples were then embedded in a paraffin block, and sections of 3 to 5 µm thickness were prepared utilizing a Leica RM 2245 microtome (Leica Microsystems, Heidelberg, Germany). For deparaffinization, the slides were placed into xylene, followed by rehydration through a descending series of alcohol solutions (from 100 to 70%, 5 min each). Subsequently, the slides were exposed to a 45-min boiling process in a 10 mM citrate buffer (Tri-sodium citrate (dihydrate) 2.94 g in 1 L distilled water, pH adjusted to 6.00 with 1.0 N HCl) at 95 °C for antigen retrieval. Following this, the sections were permeabilized in 0,5% Triton X-100 in PBS with Ca/Mg and outlined with a PAP pen (Kisker Biotech GmbH & Co. KG, Steinfurt, Germany) to create a hydrophobic barrier. The slides underwent a 30-min blocking step at room temperature in PBS with 5% goat serum, followed by overnight incubation at 4 °C or one hour incubation at 37 °C with primary antibodies raised against claudin-1, -3, -5, -7, occludin, TNFR-1, and TNR-2 (same as mentioned above). After several washing steps, the slides were then incubated with the secondary antibodies (goat anti-rabbit Alexa Fluor-488, cat. #7074; goat anti-mouse Alexa Fluor-594, cat. #7076; 1:1000, Cell Signaling Technology, Danvers, MA, USA) and DAPI for staining the cell nuclei at 37 °C for one hour. Afterwards, the tissue sections were mounted with ProTaqs Mount Fluor (Biocyc, Luckenwalde, Germany) and examined using a Zeiss 710 confocal laser scanning microscope (Zeiss, Oberkochen, Germany). We optically localized follicle-associated epithelium within the dome region of PP, which can be done with high accuracy during confocal laser scanning microscopy.

### 4.8. Vitality Test

Due to the lack of appropriate vitality tests for Peyer’s patch tissue, various substances were tested to assess the vitality of the tissue. This was performed in a pre-experiment without adding any substances to the tissue. Forskolin (5 µM), lithium carbonate (10 mM), and barium chloride (1 mM) were added to the serosal side of the tissue after 3 h in the Ussing chamber. Lithium carbonate (5, 10 mM) and barium chloride (10 mM) were added to both the serosal and mucosal side after 10 h in the Ussing chamber. The TER was then recorded for 20 min.

Finally, 25 mM of lithium carbonate added to both sides of the chamber, resulting in a final concentration of 5 mM, was used in the experiments to assess the vitality. In addition, due to the limitation of very low TER values—another factor—a cut-off TER for vital tissue [39] was taken into account.

### 4.9. Statistical Analysis

Experimental data are presented as means ± standard error of the mean (SEM) and were statistically analyzed using JMP Pro 16 software. The number of animals is specified as *n*. The significance among different groups was compared using the non-parametric Kruskal–Wallis and the Wilcoxon–Test, and *p*-values below 0.05 were considered statistically significant.

## 5. Conclusions

In this study, the ex vivo experiments validated the barrier-strengthening effect of CBD by enhancing sealing TJ proteins and reducing the expression of TNFR-1 in the porcine Peyer’s patch. Since TNFR-1 plays a crucial role in promoting inflammatory diseases, CBD could emerge as a promising drug for preventative use or as part of multimodal therapeutic approaches in the treatment of IBDs.

## Figures and Tables

**Table 1:** Buffer.

Substance	Molecular Formula	M g·mol^−1^	c_n_ mmol·L^−1^	c_m_ g·L^−1^
Sodium chloride	NaCl	58.44	119	6.95
Sodium bicarbonate	NaHCO_3_	84.01	25	2.10
Sodium dihydrogen phosphate	NaH_2_PO_4_ · H_2_O	137.99	0.6	0.08
Di-sodium hydrogen phosphate	Na_2_HPO_4_ · 2 H_2_O	177.99	2.4	0.43
Potassium chloride	KCl	74.56	5	0.37
HEPES	C_8_H_18_N_2_O_4_S	238.30	10	2.38
Glucose	C_6_H_12_O_6_·H_2_O	198.18	10	1.98
Calcium chloride	CaCl_2_ ·2 H_2_O	147.02	1.2	0.18
Magnesium chloride	MgCl_2_ · 6 H_2_O	203.30	1.2	0.24

## Data Availability

All original data are available on request.

## Abbreviations

The following abbreviations are used in this manuscript:

CBD	Cannabidiol
CBR	Cannabinoid receptor
EMA	European Medicines Agency
FAE	follicle-associated epithelium
FDA	Food and Drug Administration
GALT	gut-associated lymphoid tissue
IBDs	Inflammatory Bowel Diseases
Isc	short-current circuit
LPS	lipopolysaccharide
PP	Peyer’s Patch
SEM	standard error of the mean
TER	Transepithelial resistance
TJ	Tight junction
TNFɑ	Tumor necrosis factor alpha
TNFR	Tumor necrosis factor–receptor
VE	villus epithelium
ZO-1	Zonula occludens 1
